# [2-(2-Chloro­phen­yl)-2-hy­droxy­eth­yl](isoprop­yl)ammonium 4-hy­droxy­benzoate

**DOI:** 10.1107/S1600536810051536

**Published:** 2010-12-18

**Authors:** Ling Zhou, Yang Guang Qi, Ge Zhang, Yu Yun Xu, Hai Feng

**Affiliations:** a College of Pharmaceutical Sciences, Zhejiang University of Technology, Hangzhou 310014, People’s Republic of China

## Abstract

The title molecular salt, C_11_H_17_ClNO^+^·C_7_H_5_O_3_^−^, was obtained by the reaction of racemic clorprenaline and 4-hy­droxy­benzoic acid. In the crystal, the components are connected by O—H⋯O and N—H⋯O hydrogen bonds, resulting in a two-dimensional hydrogen-bonded network.

## Related literature

For related structures, see: Takwale & Pant (1971 ▶); Tang *et al.* (2009 ▶). For hydrogen bonding, see: Feng *et al.* (2010 ▶).
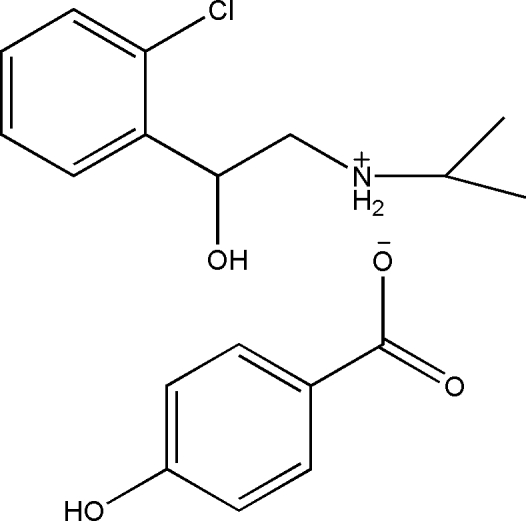


## Experimental

### 

#### Crystal data

C_11_H_17_ClNO^+^·C_7_H_5_O_3_^−^
*M* *_r_* = 351.82
Monoclinic, 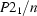

*a* = 9.4033 (4) Å
*b* = 12.2591 (4) Å
*c* = 15.9290 (7) Å
β = 96.144 (1)°
*V* = 1825.68 (13) Å^3^
*Z* = 4
Mo *K*α radiation
μ = 0.23 mm^−1^
*T* = 296 K
0.50 × 0.38 × 0.21 mm

#### Data collection

Rigaku R-AXIS RAPID/ZJUG diffractometer
Absorption correction: multi-scan (*ABSCOR*; Higashi, 1995 ▶) *T*_min_ = 0.894, *T*_max_ = 0.953
17614 measured reflections
4131 independent reflections
2891 reflections with *I* > 2σ(*I*)
*R*_int_ = 0.025

#### Refinement

*R*[*F* ^2^ > 2σ(*F* ^2^)] = 0.041
*wR*(*F* ^2^) = 0.110
*S* = 1.00
4131 reflections
219 parameters
H-atom parameters constrained
Δρ_max_ = 0.34 e Å^−3^
Δρ_min_ = −0.48 e Å^−3^

### 

Data collection: *PROCESS-AUTO* (Rigaku/MSC, 2006) ▶; cell refinement: *PROCESS-AUTO* ▶; data reduction: *CrystalStructure* (Rigaku/MSC, 2007) ▶; program(s) used to solve structure: *SHELXS97* (Sheldrick, 2008 ▶); program(s) used to refine structure: *SHELXL97* (Sheldrick, 2008 ▶); molecular graphics: *ORTEP-3 for Windows* (Farrugia, 1997 ▶); software used to prepare material for publication: *WinGX* (Farrugia, 1999 ▶).

## Supplementary Material

Crystal structure: contains datablocks global, I. DOI: 10.1107/S1600536810051536/ds2076sup1.cif

Structure factors: contains datablocks I. DOI: 10.1107/S1600536810051536/ds2076Isup2.hkl

Additional supplementary materials:  crystallographic information; 3D view; checkCIF report

## Figures and Tables

**Table 1:** Hydrogen-bond geometry (Å, °)

*D*—H⋯*A*	*D*—H	H⋯*A*	*D*⋯*A*	*D*—H⋯*A*
N1—H1*B*⋯O2	0.90	1.91	2.8090 (18)	177
N1—H1*A*⋯O3^i^	0.90	1.87	2.7671 (18)	178
O1—H101⋯O2^i^	0.82	1.94	2.7568 (16)	174
O4—H401⋯O3^ii^	0.82	1.86	2.6601 (18)	165

Symmetry codes: (i) 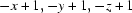; (ii) 
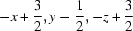
.

**Fig. 1.**
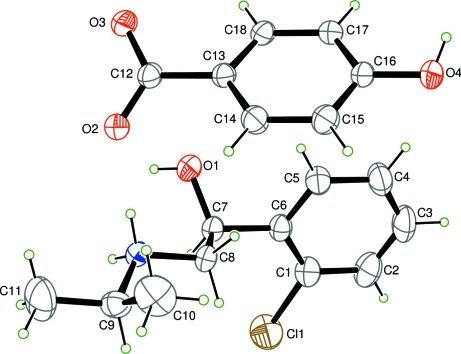
The cell unit of (I) with atom labels, showing 50% probability displacement ellipsoids.

## supplementary crystallographic information

### Comment

A recent study reports the structure of *N*-[2-(2-chlorophenyl)-2-hydroxyethyl]- propan-2-aminium-4-methylbenzoate (Feng *et al.*, 2010), which was synthesized by *p*-Toluic acid and clorprenaline (Tang *et al.*, 2009). In the present study, reaction of 4-Hydroxybenzoic acid instead of *p*-Toluic acid with racemic clorprenaline yields the title compound, (I) following a similar synthetic procedure.

In (I), the clorprenaline molecule and the 4-Hydroxybenzoic acid molecule are linked to each other by the N—H···O and the O—H···O hydogen bonds (Fig. 1 & Table 1). The clorprenaline in (I) are twisted moderately as compared with those of other compounds. The C(12)-O(2) distance of 1.257 (2)Å is much shorter than the similar distance of 1.292 (8)Å (Takwale *et al.*, 1971). The C(9)-N(1) distance of 1.509 (2)Å is longer than the value of the similar bond distance of 1.473 (4)Å (Tang *et al.*, 2009*b*) and comparable to the similar bond distance of 1.503 (2)Å (Feng *et al.*, 2010). The C(1)—C(6)—C(7)—C(8) torsion angle of 95.72 (19)° is larger than the value of the C(7)—C(2)—C(1)—C(8) torsion angle of 91.9 (2)° (Tang *et al.*, 2009).

### Experimental

Racemic clorprenaline was prepared from clorprenaline hydrochloride purchased from ShangHai Shengxin Medicine & Chemical Co., Ltd. ShangHai, China. Clorprenaline hydrochloride and NaOH in a molar ratio of 1:1 were mixed and dissolved in a methanol-water solution (1:1 *v*/*v*). The precipitate formed was filtered off, washed with water and dried. It was used without further purification. Racemic clorprenaline (0.5 g, 0.0023 mol) was dissolved in methanol (6 ml) and then 4-Hydroxybenzoic acid (0.29 g, 0.0023 mol) was added.The mixture was dissolved by stirring for 1h at room temperature. The resulting solution was concentrated at ambient temperature. Colorless crystals of (I) were separated from the solution in about 68% yield after two day.

### Refinement

All of the H atoms were placed in calculated positions and allowed to ride on their parent atoms at distances of 0.93 (aromatic), 0.98 (methine), 0.97 (methylene), 0.96 (methyl) 0.82 (hydroxyl) and N—H=0.90 Å, with *U*_iso_(H) = 1.2–1.5 *U*_eq_(C).

### Crystal data

**Table:**

C_11_H_17_ClNO^+^·C_7_H_5_O_3_^−^	*F*(000) = 744
*M**_r_* = 351.82	*D*_x_ = 1.280 Mg m^−^^3^
Monoclinic, *P*2_1_/*n*	Mo *K*α radiation, λ = 0.71073 Å
Hall symbol: -P 2yn	Cell parameters from 12291 reflections
*a* = 9.4033 (4) Å	θ = 3.1–27.4°
*b* = 12.2591 (4) Å	µ = 0.23 mm^−^^1^
*c* = 15.9290 (7) Å	*T* = 296 K
β = 96.144 (1)°	Chunk, colorless
*V* = 1825.68 (13) Å^3^	0.50 × 0.38 × 0.21 mm
*Z* = 4	

### Data collection

**Table:**

Rigaku R-AXIS RAPID/ZJUG diffractometer	4131 independent reflections
Radiation source: rolling anode	2891 reflections with *I* > 2σ(*I*)
graphite	*R*_int_ = 0.025
Detector resolution: 10.00 pixels mm^-1^	θ_max_ = 27.4°, θ_min_ = 3.1°
ω scans	*h* = −12→12
Absorption correction: multi-scan (*ABSCOR*; Higashi, 1995)	*k* = −15→15
*T*_min_ = 0.894, *T*_max_ = 0.953	*l* = −20→20
17614 measured reflections	

### Refinement

**Table:**

Refinement on *F*^2^	Primary atom site location: structure-invariant direct methods
Least-squares matrix: full	Secondary atom site location: difference Fourier map
*R*[*F*^2^ > 2σ(*F*^2^)] = 0.041	Hydrogen site location: inferred from neighbouring sites
*wR*(*F*^2^) = 0.110	H-atom parameters constrained
*S* = 1.00	*w* = 1/[σ^2^(*F*_o_^2^) + (0.0398*P*)^2^ + 0.8632*P*] where *P* = (*F*_o_^2^ + 2*F*_c_^2^)/3
4131 reflections	(Δ/σ)_max_ < 0.001
219 parameters	Δρ_max_ = 0.34 e Å^−^^3^
0 restraints	Δρ_min_ = −0.48 e Å^−^^3^

### Special details

**Table:**

Geometry. All e.s.d.'s (except the e.s.d. in the dihedral angle between two l.s. planes) are estimated using the full covariance matrix. The cell e.s.d.'s are taken into account individually in the estimation of e.s.d.'s in distances, angles and torsion angles; correlations between e.s.d.'s in cell parameters are only used when they are defined by crystal symmetry. An approximate (isotropic) treatment of cell e.s.d.'s is used for estimating e.s.d.'s involving l.s. planes.
Refinement. Refinement of *F*^2^ against ALL reflections. The weighted *R*-factor *wR* and goodness of fit *S* are based on *F*^2^, conventional *R*-factors *R* are based on *F*, with *F* set to zero for negative *F*^2^. The threshold expression of *F*^2^ > σ(*F*^2^) is used only for calculating *R*-factors(gt) *etc*. and is not relevant to the choice of reflections for refinement. *R*-factors based on *F*^2^ are statistically about twice as large as those based on *F*, and *R*- factors based on ALL data will be even larger.

### Fractional atomic coordinates and isotropic or equivalent isotropic displacement parameters (Å^2^)

**Table:**

	*x*	*y*	*z*	*U*_iso_*/*U*_eq_	
Cl1	0.62065 (7)	0.20187 (5)	0.20512 (3)	0.0746 (2)	
O2	0.41212 (13)	0.41383 (9)	0.58832 (8)	0.0428 (3)	
O3	0.59219 (14)	0.46948 (9)	0.67954 (8)	0.0462 (3)	
O4	0.72015 (17)	−0.03918 (9)	0.68325 (9)	0.0606 (4)	
H401	0.7879	−0.0416	0.7202	0.091*	
O1	0.64832 (15)	0.37003 (10)	0.44750 (9)	0.0502 (3)	
H101	0.6332	0.4338	0.4338	0.075*	
N1	0.34605 (15)	0.35386 (10)	0.41842 (9)	0.0364 (3)	
H1A	0.3652	0.4102	0.3852	0.044*	
H1B	0.3687	0.3748	0.4723	0.044*	
C17	0.7690 (2)	0.15336 (13)	0.68657 (10)	0.0398 (4)	
H17	0.8653	0.1407	0.7036	0.048*	
C13	0.57588 (18)	0.28014 (12)	0.64942 (10)	0.0352 (4)	
C6	0.68166 (18)	0.20139 (13)	0.37627 (11)	0.0387 (4)	
C7	0.58728 (18)	0.30067 (13)	0.38234 (11)	0.0377 (4)	
H7	0.5787	0.3401	0.3285	0.045*	
C8	0.44059 (18)	0.26082 (12)	0.40065 (11)	0.0379 (4)	
H8A	0.3967	0.2201	0.3525	0.045*	
H8B	0.4511	0.2122	0.4489	0.045*	
C12	0.52260 (19)	0.39509 (12)	0.63789 (10)	0.0364 (4)	
C18	0.71869 (19)	0.25922 (13)	0.67590 (10)	0.0373 (4)	
H18	0.7817	0.3172	0.6867	0.045*	
C16	0.6755 (2)	0.06630 (13)	0.67183 (11)	0.0419 (4)	
C1	0.7039 (2)	0.15117 (15)	0.30082 (12)	0.0475 (4)	
C5	0.7444 (2)	0.15316 (15)	0.45040 (13)	0.0485 (4)	
H5	0.7312	0.1846	0.5021	0.058*	
C9	0.18745 (19)	0.33141 (15)	0.40517 (12)	0.0459 (4)	
H9	0.1636	0.3057	0.3471	0.055*	
C14	0.4843 (2)	0.19169 (14)	0.63267 (12)	0.0451 (4)	
H14	0.3886	0.2039	0.6138	0.054*	
C15	0.5339 (2)	0.08564 (14)	0.64370 (13)	0.0499 (5)	
H15	0.4715	0.0274	0.6321	0.060*	
C4	0.8256 (2)	0.05966 (16)	0.44857 (16)	0.0592 (6)	
H4	0.8649	0.0280	0.4988	0.071*	
C2	0.7882 (2)	0.05865 (17)	0.29803 (16)	0.0620 (6)	
H2	0.8036	0.0276	0.2465	0.074*	
C3	0.8488 (2)	0.01326 (17)	0.37280 (18)	0.0661 (6)	
H3	0.9053	−0.0489	0.3718	0.079*	
C10	0.1482 (2)	0.2436 (2)	0.46424 (17)	0.0748 (7)	
H10A	0.2010	0.1784	0.4549	0.112*	
H10B	0.0476	0.2288	0.4540	0.112*	
H10C	0.1709	0.2673	0.5215	0.112*	
C11	0.1090 (3)	0.4372 (2)	0.4154 (2)	0.0839 (8)	
H11A	0.1381	0.4901	0.3761	0.126*	
H11B	0.1313	0.4639	0.4719	0.126*	
H11C	0.0079	0.4251	0.4046	0.126*	

### Atomic displacement parameters (Å^2^)

**Table:**

	*U*^11^	*U*^22^	*U*^33^	*U*^12^	*U*^13^	*U*^23^
Cl1	0.0952 (5)	0.0856 (4)	0.0451 (3)	0.0206 (3)	0.0172 (3)	0.0027 (3)
O2	0.0505 (7)	0.0338 (6)	0.0419 (7)	0.0054 (5)	−0.0060 (6)	−0.0023 (5)
O3	0.0609 (8)	0.0268 (6)	0.0473 (7)	−0.0027 (5)	−0.0103 (6)	−0.0025 (5)
O4	0.0820 (11)	0.0254 (6)	0.0667 (10)	0.0029 (6)	−0.0273 (8)	−0.0004 (6)
O1	0.0550 (8)	0.0324 (6)	0.0602 (8)	−0.0013 (6)	−0.0078 (6)	−0.0053 (6)
N1	0.0410 (8)	0.0314 (7)	0.0366 (7)	0.0006 (6)	0.0029 (6)	−0.0031 (6)
C17	0.0463 (10)	0.0329 (8)	0.0377 (9)	0.0015 (7)	−0.0069 (7)	−0.0017 (7)
C13	0.0452 (10)	0.0277 (7)	0.0317 (8)	−0.0013 (7)	−0.0004 (7)	0.0013 (6)
C6	0.0362 (9)	0.0330 (8)	0.0478 (10)	−0.0001 (7)	0.0083 (7)	0.0011 (7)
C7	0.0426 (10)	0.0292 (8)	0.0409 (9)	0.0018 (7)	0.0033 (7)	0.0008 (7)
C8	0.0439 (10)	0.0257 (7)	0.0440 (9)	0.0014 (7)	0.0044 (7)	−0.0013 (7)
C12	0.0461 (10)	0.0297 (8)	0.0333 (8)	−0.0007 (7)	0.0035 (7)	0.0009 (6)
C18	0.0456 (10)	0.0276 (8)	0.0372 (9)	−0.0044 (7)	−0.0020 (7)	−0.0010 (6)
C16	0.0589 (11)	0.0255 (8)	0.0384 (9)	0.0002 (7)	−0.0083 (8)	0.0015 (6)
C1	0.0470 (11)	0.0449 (10)	0.0527 (11)	0.0026 (8)	0.0147 (9)	0.0029 (8)
C5	0.0493 (11)	0.0415 (10)	0.0539 (11)	0.0042 (8)	0.0023 (9)	0.0009 (8)
C9	0.0372 (10)	0.0539 (11)	0.0459 (10)	−0.0020 (8)	0.0017 (8)	−0.0062 (8)
C14	0.0439 (10)	0.0347 (9)	0.0543 (11)	−0.0052 (7)	−0.0062 (8)	0.0043 (8)
C15	0.0568 (12)	0.0294 (8)	0.0605 (12)	−0.0104 (8)	−0.0088 (9)	0.0036 (8)
C4	0.0524 (12)	0.0464 (11)	0.0770 (15)	0.0114 (9)	−0.0012 (11)	0.0074 (10)
C2	0.0587 (13)	0.0552 (12)	0.0765 (15)	0.0088 (10)	0.0275 (12)	−0.0125 (11)
C3	0.0521 (13)	0.0443 (11)	0.1031 (19)	0.0147 (9)	0.0138 (13)	−0.0011 (12)
C10	0.0536 (13)	0.0820 (16)	0.0902 (19)	−0.0165 (12)	0.0137 (13)	0.0151 (14)
C11	0.0480 (13)	0.0737 (16)	0.131 (2)	0.0158 (12)	0.0143 (15)	−0.0056 (16)

### Geometric parameters (Å, °)

**Table:**

Cl1—C1	1.751 (2)	C8—H8B	0.9700
O2—C12	1.257 (2)	C18—H18	0.9300
O3—C12	1.2678 (19)	C16—C15	1.379 (3)
O4—C16	1.3656 (19)	C1—C2	1.387 (3)
O4—H401	0.8200	C5—C4	1.380 (3)
O1—C7	1.415 (2)	C5—H5	0.9300
O1—H101	0.8200	C9—C10	1.502 (3)
N1—C8	1.492 (2)	C9—C11	1.510 (3)
N1—C9	1.509 (2)	C9—H9	0.9800
N1—H1A	0.9000	C14—C15	1.386 (2)
N1—H1B	0.9000	C14—H14	0.9300
C17—C18	1.385 (2)	C15—H15	0.9300
C17—C16	1.387 (2)	C4—C3	1.372 (3)
C17—H17	0.9300	C4—H4	0.9300
C13—C18	1.388 (2)	C2—C3	1.381 (3)
C13—C14	1.392 (2)	C2—H2	0.9300
C13—C12	1.500 (2)	C3—H3	0.9300
C6—C1	1.386 (3)	C10—H10A	0.9600
C6—C5	1.394 (3)	C10—H10B	0.9600
C6—C7	1.515 (2)	C10—H10C	0.9600
C7—C8	1.521 (2)	C11—H11A	0.9600
C7—H7	0.9800	C11—H11B	0.9600
C8—H8A	0.9700	C11—H11C	0.9600
			
C16—O4—H401	109.5	C6—C1—Cl1	120.15 (14)
C7—O1—H101	109.5	C2—C1—Cl1	117.77 (16)
C8—N1—C9	115.73 (13)	C4—C5—C6	121.31 (19)
C8—N1—H1A	108.3	C4—C5—H5	119.3
C9—N1—H1A	108.3	C6—C5—H5	119.3
C8—N1—H1B	108.3	C10—C9—C11	113.15 (19)
C9—N1—H1B	108.3	C10—C9—N1	110.40 (16)
H1A—N1—H1B	107.4	C11—C9—N1	108.36 (16)
C18—C17—C16	119.89 (16)	C10—C9—H9	108.3
C18—C17—H17	120.1	C11—C9—H9	108.3
C16—C17—H17	120.1	N1—C9—H9	108.3
C18—C13—C14	118.20 (15)	C15—C14—C13	120.90 (17)
C18—C13—C12	120.69 (14)	C15—C14—H14	119.5
C14—C13—C12	121.09 (15)	C13—C14—H14	119.5
C1—C6—C5	117.18 (16)	C16—C15—C14	120.15 (16)
C1—C6—C7	123.76 (16)	C16—C15—H15	119.9
C5—C6—C7	118.97 (16)	C14—C15—H15	119.9
O1—C7—C6	109.57 (14)	C3—C4—C5	120.2 (2)
O1—C7—C8	110.94 (14)	C3—C4—H4	119.9
C6—C7—C8	107.68 (13)	C5—C4—H4	119.9
O1—C7—H7	109.5	C3—C2—C1	119.1 (2)
C6—C7—H7	109.5	C3—C2—H2	120.5
C8—C7—H7	109.5	C1—C2—H2	120.5
N1—C8—C7	111.22 (13)	C4—C3—C2	120.11 (19)
N1—C8—H8A	109.4	C4—C3—H3	119.9
C7—C8—H8A	109.4	C2—C3—H3	119.9
N1—C8—H8B	109.4	C9—C10—H10A	109.5
C7—C8—H8B	109.4	C9—C10—H10B	109.5
H8A—C8—H8B	108.0	H10A—C10—H10B	109.5
O2—C12—O3	122.88 (15)	C9—C10—H10C	109.5
O2—C12—C13	119.43 (14)	H10A—C10—H10C	109.5
O3—C12—C13	117.69 (15)	H10B—C10—H10C	109.5
C17—C18—C13	121.13 (15)	C9—C11—H11A	109.5
C17—C18—H18	119.4	C9—C11—H11B	109.5
C13—C18—H18	119.4	H11A—C11—H11B	109.5
O4—C16—C15	118.57 (15)	C9—C11—H11C	109.5
O4—C16—C17	121.75 (17)	H11A—C11—H11C	109.5
C15—C16—C17	119.67 (15)	H11B—C11—H11C	109.5
C6—C1—C2	122.05 (19)		
			
C1—C6—C7—O1	−143.52 (17)	C7—C6—C1—C2	−178.28 (18)
C5—C6—C7—O1	40.1 (2)	C5—C6—C1—Cl1	176.36 (14)
C1—C6—C7—C8	95.72 (19)	C7—C6—C1—Cl1	−0.1 (2)
C5—C6—C7—C8	−80.64 (19)	C1—C6—C5—C4	0.3 (3)
C9—N1—C8—C7	157.85 (14)	C7—C6—C5—C4	176.88 (17)
O1—C7—C8—N1	52.93 (18)	C8—N1—C9—C10	62.5 (2)
C6—C7—C8—N1	172.83 (14)	C8—N1—C9—C11	−173.03 (18)
C18—C13—C12—O2	155.55 (16)	C18—C13—C14—C15	1.2 (3)
C14—C13—C12—O2	−23.4 (3)	C12—C13—C14—C15	−179.77 (17)
C18—C13—C12—O3	−24.7 (2)	O4—C16—C15—C14	178.98 (18)
C14—C13—C12—O3	156.32 (17)	C17—C16—C15—C14	−1.9 (3)
C16—C17—C18—C13	−0.9 (3)	C13—C14—C15—C16	0.2 (3)
C14—C13—C18—C17	−0.9 (3)	C6—C5—C4—C3	1.3 (3)
C12—C13—C18—C17	−179.87 (15)	C6—C1—C2—C3	1.8 (3)
C18—C17—C16—O4	−178.65 (17)	Cl1—C1—C2—C3	−176.45 (17)
C18—C17—C16—C15	2.3 (3)	C5—C4—C3—C2	−1.4 (3)
C5—C6—C1—C2	−1.9 (3)	C1—C2—C3—C4	−0.1 (3)

### Hydrogen-bond geometry (Å, °)

**Table:**

*D*—H···*A*	*D*—H	H···*A*	*D*···*A*	*D*—H···*A*
N1—H1B···O2	0.90	1.91	2.8090 (18)	177
N1—H1A···O3^i^	0.90	1.87	2.7671 (18)	178
O1—H101···O2^i^	0.82	1.94	2.7568 (16)	174
O4—H401···O3^ii^	0.82	1.86	2.6601 (18)	165

Symmetry codes: (i) −*x*+1, −*y*+1, −*z*+1; (ii) −*x*+3/2, *y*−1/2, −*z*+3/2.
